# Successful Treatment of Refractory and Relapsed CNS Acute Lymphoblastic Leukemia With CD-19 CAR-T Immunotherapy: A Case Report

**DOI:** 10.3389/fonc.2021.699946

**Published:** 2021-08-26

**Authors:** Kyaw Thu Htun, Qiang Gong, Le Ma, Ping Wang, Ya Tan, Guangsheng Wu, Jieping Chen

**Affiliations:** ^1^Department of Hematology, Southwest Hospital, First Affiliated Hospital of the Army Medical University, Chongqing, China; ^2^Hematology Department, First Affiliated Hospital of Shihezi University, Shihezi, China

**Keywords:** acute lymphoblastic leukemia, central nervous system, refractory and relapsed, car-t, case report

## Abstract

In recent decades, survival was significantly improved in B cell acute lymphoblastic leukemia (B-ALL) patients. But refractory and relapsed B-ALL still has aggressive clinical behavior and poor prognosis. Especially, the patients with central nervous system infiltration is very difficult to achieve complete remissions with routine treatment. Chimeric antigen receptor-modified T-cell therapy targeting CD-19 has shown to be a beneficial treatment approach in refractory and relapsed B cell acute lymphoblastic leukemia (r/r ALL). However, there are very few studies reporting to treatment of refractory and relapsed B cell ALL with central nervous system infiltration. Here, we reported one single case of a patient diagnosed with relapsed B cell ALL with CNS infiltration who was successfully treated by second generation CAR containing a co-stimulator CD28 or 4-1BB therapy. Long-term proliferation of CAR-T cells in peripheral blood and bone marrow was observed more than 18 months. After CAR-T treatment, the patient got toxicity of grade 1 cytokine release syndrome and achieved significantly 36 months event free survival of follow-up. It is suggested that CD-19 CAR containing CD28 or 4-1BB costimulatory may be an effective therapy in refractory and relapsed B cell ALL with central nervous system infiltration. Its toxicity is mild, and its safety is high.

**Clinical Trial Registration:**ClinicalTrials.gov Identifier: NCT02349698.

## Introduction

Central nervous system leukemia is one of the most common extra-medullary ALL relapse (1). Standard chemotherapy is the first line treatment for ALL patients who has extra-medullary relapse. However, the survival of patients with CNS leukemia is particularly poor with ALL (2), which is an important cause of treatment failure among patients (3).

On recent years, chimeric antigen receptor modified T-cell (CAR-T) therapy targeting CD19 has revealed to be effective treatment approach for relapsed/refractory B cell acute lymphoblastic leukemia (4). Studies of anti-CD19 CAR-T cells in relapsed B cell acute lymphoblastic leukemia (r/r ALL) have shown high rates of durable remission (5, 6). However, there are only few studies regarding the treatment of extra-medullary r/r B cell ALL nervous system and lack of clinical experience using CAR-T cells to treat the disease. Here, we reported a single case of r/r B-ALL patient with CNS infiltration who was successfully treated by a CAR containing co-stimulator CD28 or 4-1BB treatment. We demonstrate that CAR-T cell therapy is safe and feasible for the treatment of CNS relapsed B cell acute lymphoblastic leukemia, and the remission observed lasts for 36 months.

## Case Report

### Leukemia Treatment History

A 16-year-old Chinese male patient, who was a student without any other special past medical history, presented with high fever and recurrent headache, was initially admitted to the First Affiliated Hospital of Shihezi University in Xinjiang province. He was diagnosed as B-ALL in high risk in May 2016. The bone marrow graphic analysis revealed 82% malignant naive lymphocytes. Bone morrow flow cytometry demonstrated that 83.5% karyocytes were malignant B naive lymphocytes (HLA-DR, CD10+ CD19+ CD20+ CD22+ CD38+ cCD79a, TdT, partially expressed CD34). Leukemia related fusion gene is negative. Chromosome: 46,XY, t (6, 9) (p22, p24), add (9) (q34), add (14) (q32) (CP15)/46, XY (5).

**Figure 1:**
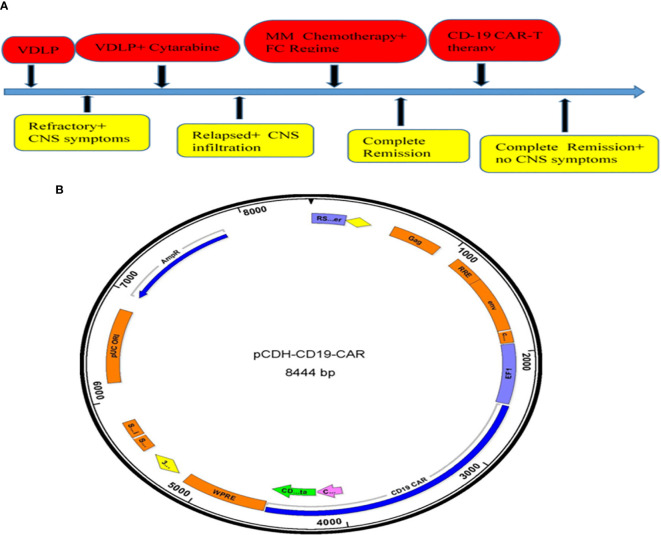
Summary protocol of medication treatment and CD19CAR structure. **(A)** Summary protocol of the patient’s treatment. **(B)** The schematic diagram of pCDH-CD19-CAR.

After one course of induction therapy with VDLP (vincristine + daunorubicin + L-asparaginase + prednisolone), bone marrow morphology still revealed 55% immature cells which mean no remission in bone marrow. At the same time, the patient suffered serious headache and diplopia with increasing cerebrospinal fluid pressure measuring about 300 mmH_2_O, and the result of cerebrospinal fluid flow cytometry revealed that 91.13% karyocytes were malignant B naive lymphocytes (**Supplementary Figure S1**), which indicated refractory B-ALL with central infiltration. Then, the patient received two cycles of inter-median dose cytarabine followed by VDLP regimen one course again (**Figure 1A**). After two cycles of inter-median dose cytarabine treatment, the result of malignant lymphocytes in his cerebrospinal fluid was 42.54% and 22.48% (**Supplementary Figure S2** and **Supplementary Figure S3**), respectively. The lumbar puncture and intrathecal injection (cytarabine 50 mg, methotrexate 10 mg, dexamethasone 5 mg) was operated after every course of chemotherapy. Finally, the patient achieved complete remission in bone marrow, but there were still 2% malignant lymphocytes in his cerebrospinal fluid (**Supplementary Figure S4**).

Subsequently, the patient was transferred to the First Affiliated Hospital of the Third Military Medical University for further management. After one course of Mm chemotherapy with intrathecal chemotherapy, the patient still maintained CR status in bone marrow, but it was tested that about 0.16% malignant lymphocytes in the cerebrospinal fluid (**Figure 3A**). Considering funding difficulties, the patient who had no choice to do allo-transplantation was enrolled to our clinical trials (Clinical Trials information: NCT02349698).

### Manufacturing of pCDH-CD19-CAR

The peripheral mononuclear cells were extracted from patient and transfected with lentivirus vector carrying CD19- CAR gene and heparin anticoagulant in GMP laboratory. CAR-T lymphocytes were cultured in GMP laboratories to collect and apply to patients under quality control. The chimeric antigen receptor CAR used in this study was a CAR containing a co-stimulator CD28 or 4-1BB. The structural composition of CAR: CD19 scFV = anti-CD19 single chain antibody, consisting of heavy chain variable region and light chain variable region, CD28 or 4-1BB, is a cytoplasmic costimulating molecule fragment of CAR; CD3zeta = active Zeta fragment of CD3 molecule (**Figure 1B**). The CAR was built on a modified pCDH lentivirus vector (plasmid derived from SBI). The carrier and its three independent plasmid expression systems, namely, the packing plasmid carrier, belong to the third generation lentivirus carrier, which is a more secure carrier system. T cells were produced by following a procedure of autologous T-cell culture and cell plate transfection, T-cell transfection and culture (days 0–8), and CAR-T cell harvesting by components of anti-CD3 monoclonal antibody OKT3 (Ortho Biotech), Retronectin: Takara, Japan, and Ficoll-Paque PLUS – GE Health Care Life Sciences. In the final infused cell products, the CAR transfection efficiency was 32.07% of CD3+ CAR+ cells.

### CAR19-T Cell Infusion and Patient’s Outcomes

**Figure 2:**
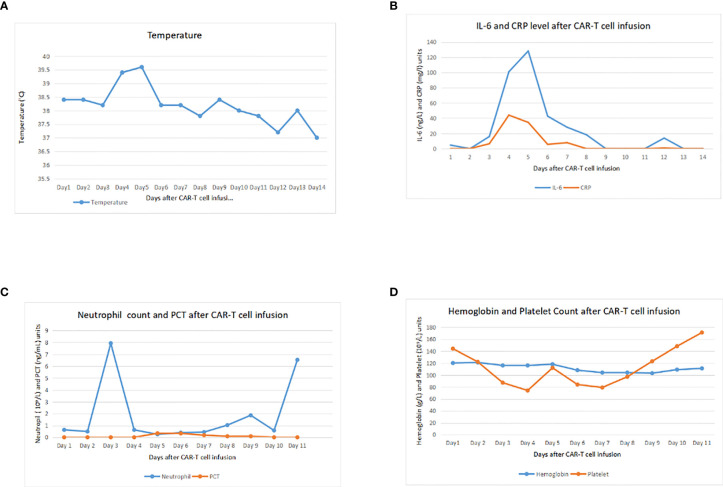
Clinical monitoring after infusion of pCDH-CD19-CAR cells. **(A)** Trends of temperature during 14 days after infusion. **(B)** Trends of interleukin (IL)-6 and C-reactive protein (CRP) during 14 days after infusion. **(C)** Changes of neutrophil count and procalcitonin (PCT) during 14 days after infusion. **(D)** Changes of hemoglobin (Hb) and platelet (PLT) during 14 days after infusion.

Before CAR-T cell infusion, the patient received conditioning chemotherapy, FC regime for 4 days with fludarabine 25 mg/m^2^ in days 1–4 and cyclophosphamide 250 mg/kg day 4. After chemotherapy, bone marrow puncture was done and the result indicated CR. Two days after lymphodepleting chemotherapy, the patient received CD-19 CAR-T cell infusion at a total dose of 2.83×106/kg for 2 consecutive days without any reactions. Blood routine and inflammatory indicators including procalcitonin, C-reactive protein, and interlukin-6 were checked every 3 days. Four days after infusion, the patient suffered tension headache (pain score is 4) accompanied by vomiting and high body temperature (**Figure 2A**) with increasing CRP and interlukin-6 level. Although head MRI and chest CT scan showed negative, ICANS and CRS could not be ruled out according to our experience. Interlukin-6 receptor antibody (tocilizumab) was immediately used for CRS, and mannitol was used for dehydration. After tocilizumab infusion, the headache was relieved quickly. CRP and interlukin-6 level upward to around 44.03 mg/L and 100.90 ng/L on next day (**Figure 2B**) and observed grade 1 cytokine release syndrome. Neutropenia (**Figure 2C**) and thrombocytopenia were observed and corrected with granulocyte colony stimulating factor but did not have significant anemia (**Figure 2D**). Liver enzymes and albumin were still constant. Seven days after infusion, fever was recovered and all screenings and biochemical parameters returned to normal.

**Figure 3:**
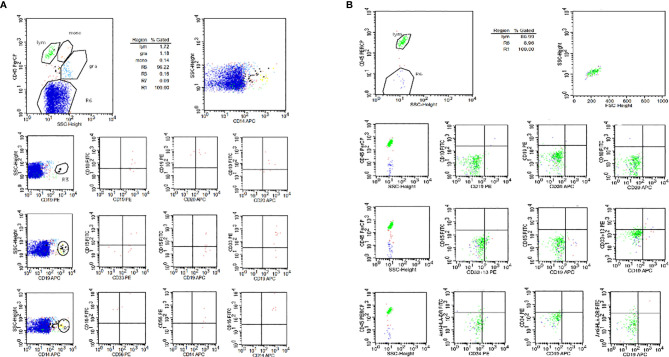
The result of cerebrospinal fluid flow cytometry. **(A)** The result of cerebrospinal fluid flow cytometry before CAR-T treatment, 0.16% malignant lymphocytes detected in CSF. **(B)** The result of cerebrospinal fluid flow cytometry after CAR-T treatment, no leukemic cells detected in CSF.

**Figure 4:**
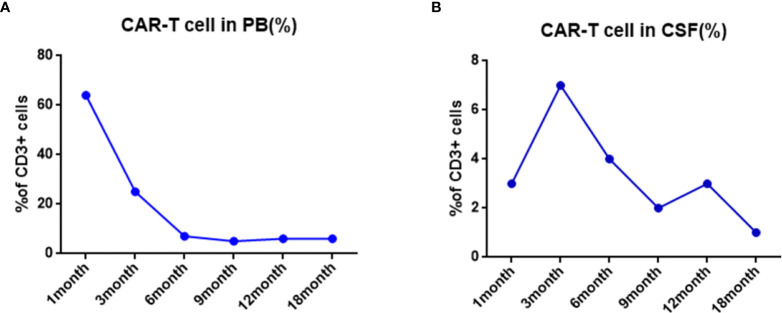
Changes of total CART percent after CAR-T treatment. **(A)** The proportion of CAR-T cells in CD3 positive T cells was detected by flow cytometry in PB. **(B)** The proportion of CAR-T cells in CD3 positive T cells was detected by flow cytometry in CSF.

One month after CAR-T cell therapy, the patient achieved complete remission and no leukemic cells in bone marrow morphology and cerebrospinal fluid (**Figure 3B**). The persistence of CAR-T cell was detected by real time polymerase chain reaction (PCR) and flow cytometry after 1 month of infusion and sustained for a long time. We tested the percentage of CAR-T cells in peripheral blood and cerebrospinal fluid by flow after CAR-T therapy in 1, 3, 6, 9, 12, and 18 months, respectively (**Figures 4A, B**). We tested blood routine and did bone marrow puncture and lumbar puncture after CAR-T therapy in 1 month, 3 months, 6 months, 1 year, 2 years, and 3 years, respectively. The result of follow-up data indicated that the patient sustained CR and even MRD was negative. Long-term proliferation of CAR-T cells in peripheral blood and bone marrow was observed more than 18 months. Fortunately, the patient got 36 months free survival with no refractory and relapse without taking any further chemotherapy or hematopoietic stem cell transplantation.

## Discussion

Acute lymphoblastic leukemia is a common malignant hematologic disease, which occurs in children and adolescents. Adult ALL accounts for about 20–30% of leukemia in adult (7). In the last decade, with the development of the diagnostic methods and treatment of ALL, the rate of overall survival (OS) and complete remission (CR) of ALL patients after treatment have increased to a great extent (8, 9). However, there were still many patients who inevitably have disease recurrence. Currently, the treatment of refractory and relapsed ALL (R/R ALL) still has great difficulty (10), especially patients with central nervous system infiltration have a worse prognosis (11). In recent years, there is no unified treatment regimen for R/R ALL with CNS infiltration. Most of centers mainly supplemented the frequency of intrathecal injection of chemotherapeutic and combine conventional chemotherapy drugs to achieve CR as far as possible.

Patients who failed standard primary and secondary chemotherapy need to lack of improving advanced therapy. CD-19 chimeric antigen receptor T cells are new novel techniques of immunotherapy in refractory or relapsed B cell acute lymphoblastic leukemia (12–14). CD-19 CAR-T cells also have one option for patients who do not respond to hematopoietic stem cell transplantation with promising results (15, 16). The therapeutic effect of CD-19 CAR-T cell ensures more benefits in central nervous system leukemia due to crossing of blood-brain-barrier (BBB) (17). Some studies showed that CD-19 CAR-T as a beneficial effect of CAR-T cell infusion on patients with active CNSL and a feasible and safe treatment against central nervous system leukemia after intrathecal chemotherapy in adult with r/r B-ALL (18, 19). All these reports suggest that CAR-T cell can enter central nervous system and eliminate leukemic cells and even promising results in extra-medullary skin and testicles with mild toxicity (20). So, we utilized CD-19 CAR-T therapy, although the patient still received complete remission in bone marrow.

According to criteria set forth by the children’s oncology group (21), CNS infiltration was observed in our case on identifying neurological symptoms, headache, diplopia, increased CSF pressure, and infiltrating white blood cells ≥5 WBCs/µl in cytology.

Prior to CAR-T cell infusion, choice of chemotherapy and further combined management was also still a debate of clinicians which had different outcomes based on multiple choice of therapies. Some chose either hematopoietic stem cell transplantation or intensive chemotherapy in accordance with individualized patients and risk assessment. We selected intra-thecal chemotherapy combined with Mm (Methotrexate, mercaptopurine) chemotherapy regime followed by Lymphodepletion therapy, FC regime.

Life-threating CRS and CRES are still main problems of CAR-T cell infusion in clinical treatment, caused by quick increasing multiple cytokines especially IL-6. These toxicities were so serious and need to control safety and monitor frequently (22–25). Cheng Qian and his colleagues reported that plasma exchange (PE) as a feasible method in management of CRS grade ≥3, by therapeutic effects which removed inflammatory factors and alleviated decreasing of CAR-T related toxicities (26). Some utilized corticosteriods to control these adverse effects, however which disturbed to long-term proliferation of CAR-T cells persistence. In our study, cytokine release syndrome grade 1 was observed according to American Society for Transplantation and Cellular Therapy (ASTCT) consensus CRS grading system and we injected only interlukin-6 receptor monoclonal antibody, tocilizumab, which was the standard procedure in clinical treatment of severe CRS.

Because toxicity management is an essential part of CAR-T cell infusion, although there are still no good solutions to overcome, we recommended to early use of tocilizumab depending on our case experience which relieved toxicity symptoms quickly and maintain long-term CAR-T cell persistence to extend event free survival. The persistence of CAR-T cell could be interrupted by high dose corticosteroid and IL-2 injections by previous studies, but neither used in our case as a difference.

Besides, serious reactions of CAR-T cell were directly related to disease tumor burden, as shown in initial studies which high toxicities of CRS grading occurred in high disease tumor burden. However, our case received grade 1 CRS only without CRES, as a being of low disease tumor burden.

Some reports mentioned that CAR-T therapy had as a bridge between hematopoietic stem cell transplantation and indicated prominent efficiency whether use of chemotherapy after CAR-T therapy (27). Further cytotoxic chemotherapy was often needed in some cases to be strengthen complete remission.

Surprisingly, the patient achieved 36 months event free survival without cytotoxic chemotherapy after CAR-T therapy and we are still following the patient closely to test for MRD. In our case study, we have two limitations. Firstly, we just have one case which lacks of enough evidence to prove the effectiveness and safety of CAR-T against r/r ALL with CNS infiltration. Secondly, the patient went home to continue his studies. Due to the lack of local medical capacity, some medical indicators of the patient could not be tested in detail, which could not explain why the efficacy of CAR-T was so good. Future clinical studies with large sample sizes are needed to clarify the effectiveness and safety of pCDH-CD19-CAR cell therapy in r/r ALL with CNS infiltration.

## Patient Perspective

I am a student with high fever and recurrent headache and went to hospital for help. The doctor told me that I was diagnosed with acute lymphoblastic leukemia through physical examinations. I felt so helpless. After the first chemotherapy cycle, my headache not only relieve but also worsened. The examination results showed that the bone marrow was in a state of no relief, and there was also infiltration in the central nervous system. I did not have enough money for a stem cell transplant. I had to continue the chemotherapy. After four rounds of chemotherapy, my bone marrow was in complete remission (CR), but there were still leukemia cells in the central nervous system. I am glad to participate in the clinical trial and has received the CAR19-T cell therapy. My central nervous system was in the stage of MRD negative, and my headache was relieved after CAR19-T cell treatment. Until now, I have maintained CR for 36 months and continued my studies. I hope that my case will give some inspiration to the patients with CNS relapsed B cell ALL all over the world.

## Data Availability Statement

The raw data supporting the conclusions of this article will be made available by the authors, without undue reservation.

## Ethics Statement

The studies involving human participants were reviewed and approved by Ethics Committee of Southwest Hospital, the First Affiliated Hospital of Third Military Medical University, Chongqing. Written informed consent to participate in this study was provided by the participants’ legal guardian/next of kin. Written informed consent was obtained from the minor(s)’ legal guardian/next of kin for the publication of any potentially identifiable images or data included in this article.

## Author Contributions

JC, QG and GW conceived and designed study. GW provided the patient’s test results. HX and SY analyzed and interpreted the data. KH and LM wrote the manuscript. LM revised the manuscript. PW, YT, and SL provide the study materials of patient. All authors contributed to the article and approved the submitted version.

## Funding

This study was supported by clinical trials (ClinicalTrials.gov number NCT02349698), Southwest Hospital, Third Military Medical University, Chongqing, China.

## Conflict of Interest

The authors declare that the research was conducted in the absence of any commercial or financial relationships that could be construed as a potential conflict of interest.

## Publisher’s Note

All claims expressed in this article are solely those of the authors and do not necessarily represent those of their affiliated organizations, or those of the publisher, the editors and the reviewers. Any product that may be evaluated in this article, or claim that may be made by its manufacturer, is not guaranteed or endorsed by the publisher.

## Supplementary Material

Supplementary Figure 1 The first result of cerebrospinal fluid flow cytometry.

Supplementary Figure 2 The first result of cerebrospinal fluid flow cytometry after one course of inter-median dose cytarabine treatment.

Supplementary Figure 3 The second result of cerebrospinal fluid flow cytometry after one course of inter-median dose cytarabine treatment.

Supplementary Figure 4 The result of cerebrospinal fluid flow cytometry after one course of VDLP regimen treatment.

## Abbreviations

ALL, acute lymphoblastic leukemia; BBB, blood brain barrier; CAR-T, chimeric antigen receptor-modified T cell; CNS, central nervous system; CNSL, central nervous system leukemia; CRP, C- reactive protein; CSF, cerebrospinal fluid; CRS, cytokine release syndrome; CRES, cytokine release encephalopathy syndrome; CSF, cerebrospinal fluid; IL-6, interleukin 6; PCR, polymerase chain reaction; r/r, refractory and relapsed.
