# Supplementary figures and images for: Successful Treatment of Refractory and Relapsed CNS Acute Lymphoblastic Leukemia With CD-19 CAR-T Immunotherapy: A Case Report
Source: Front Oncol. 2021 Aug 26;11:699946. doi: 10.3389/fonc.2021.699946 (PMC8427303; doi:10.3389/fonc.2021.699946)

Supplemental Figure1

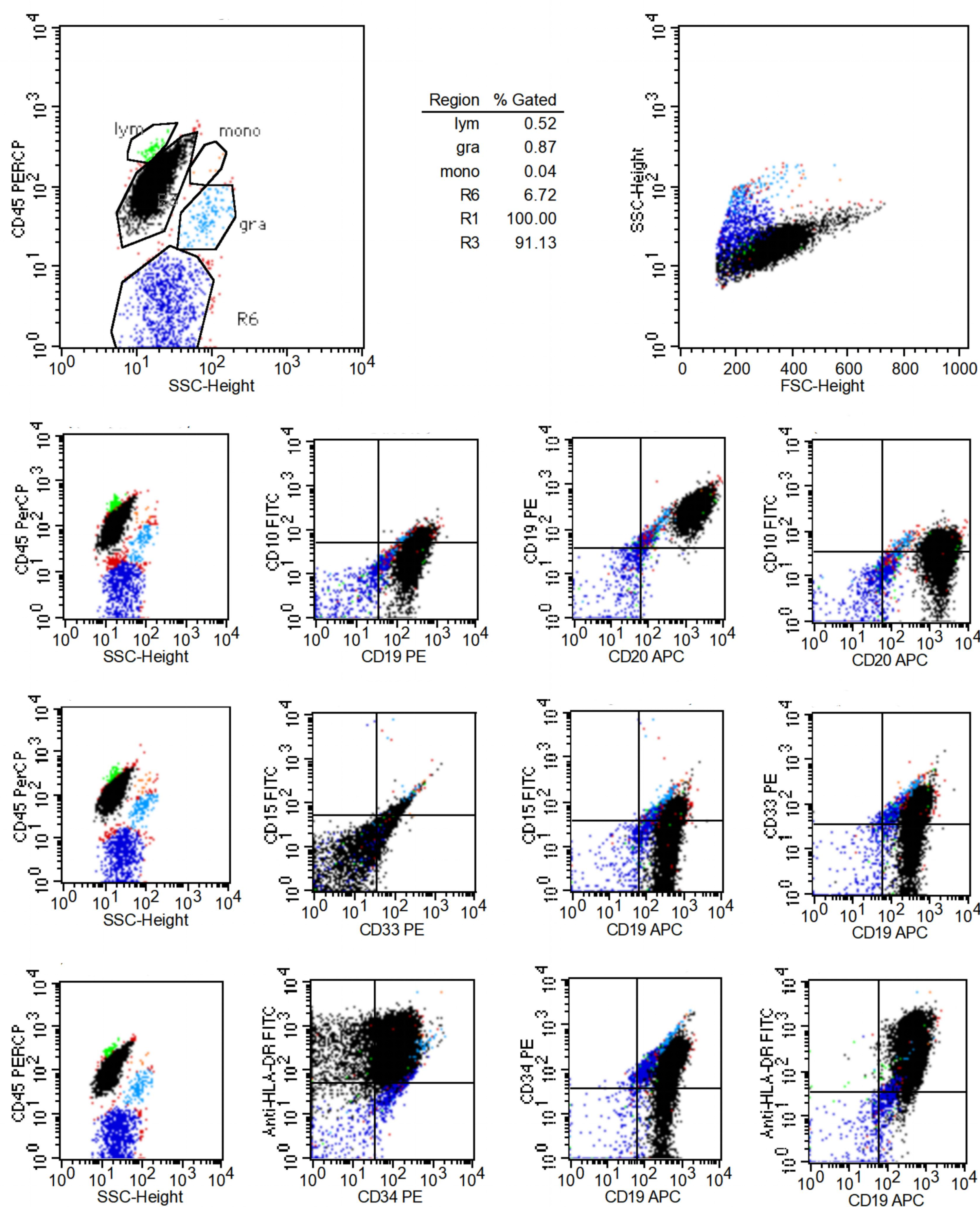

Supplemental Figure2

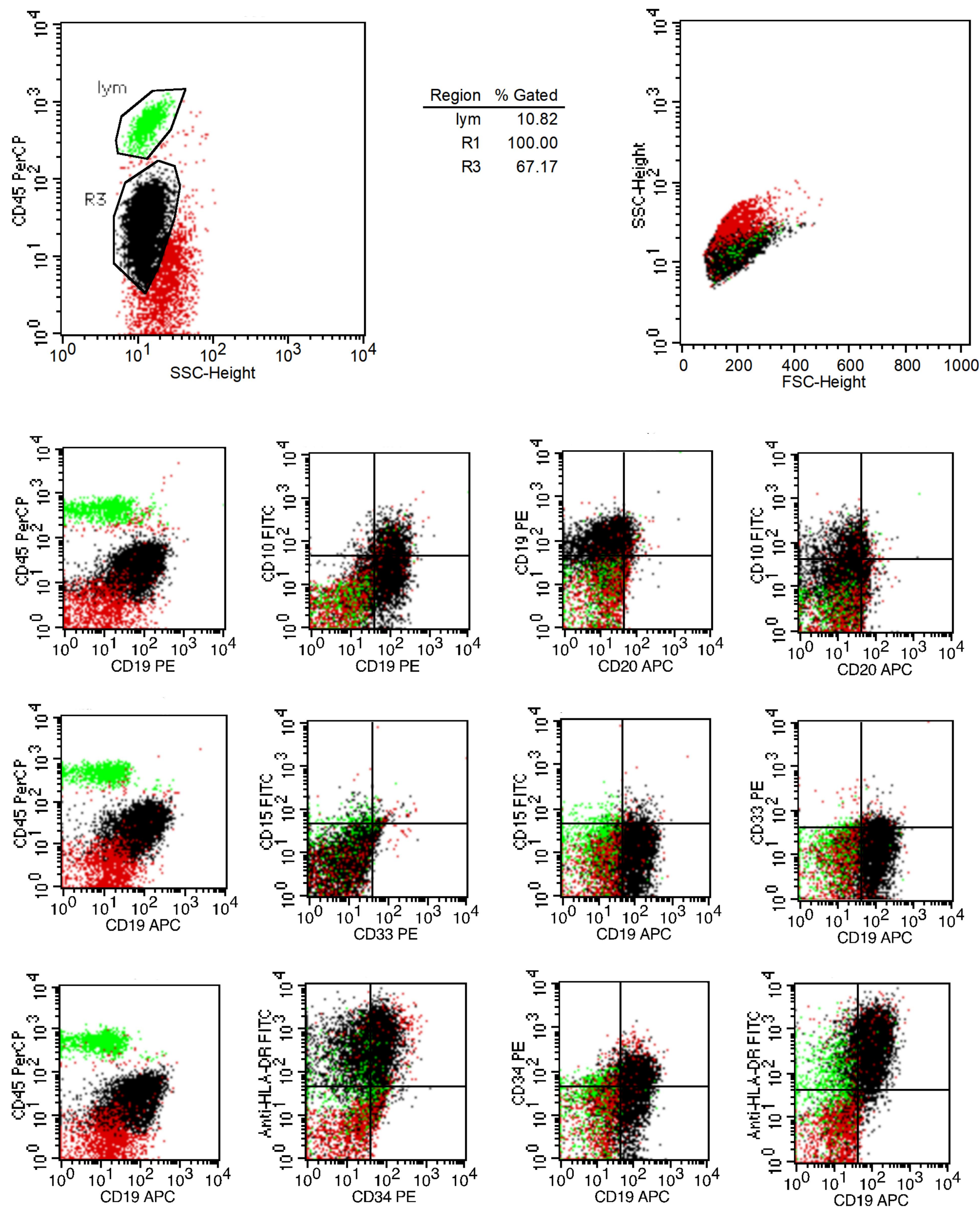

Supplemental Figure3

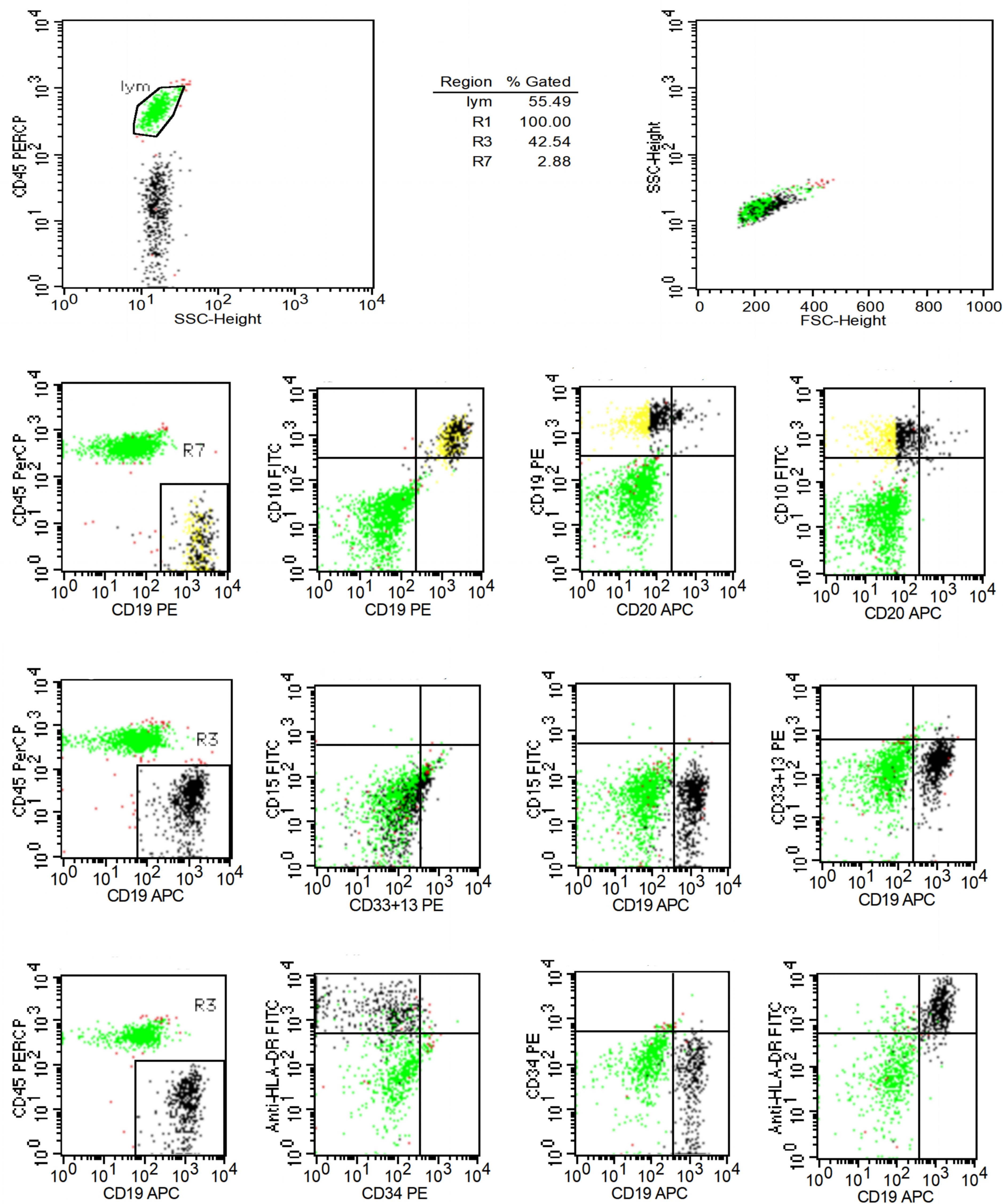

Supplemental Figure4

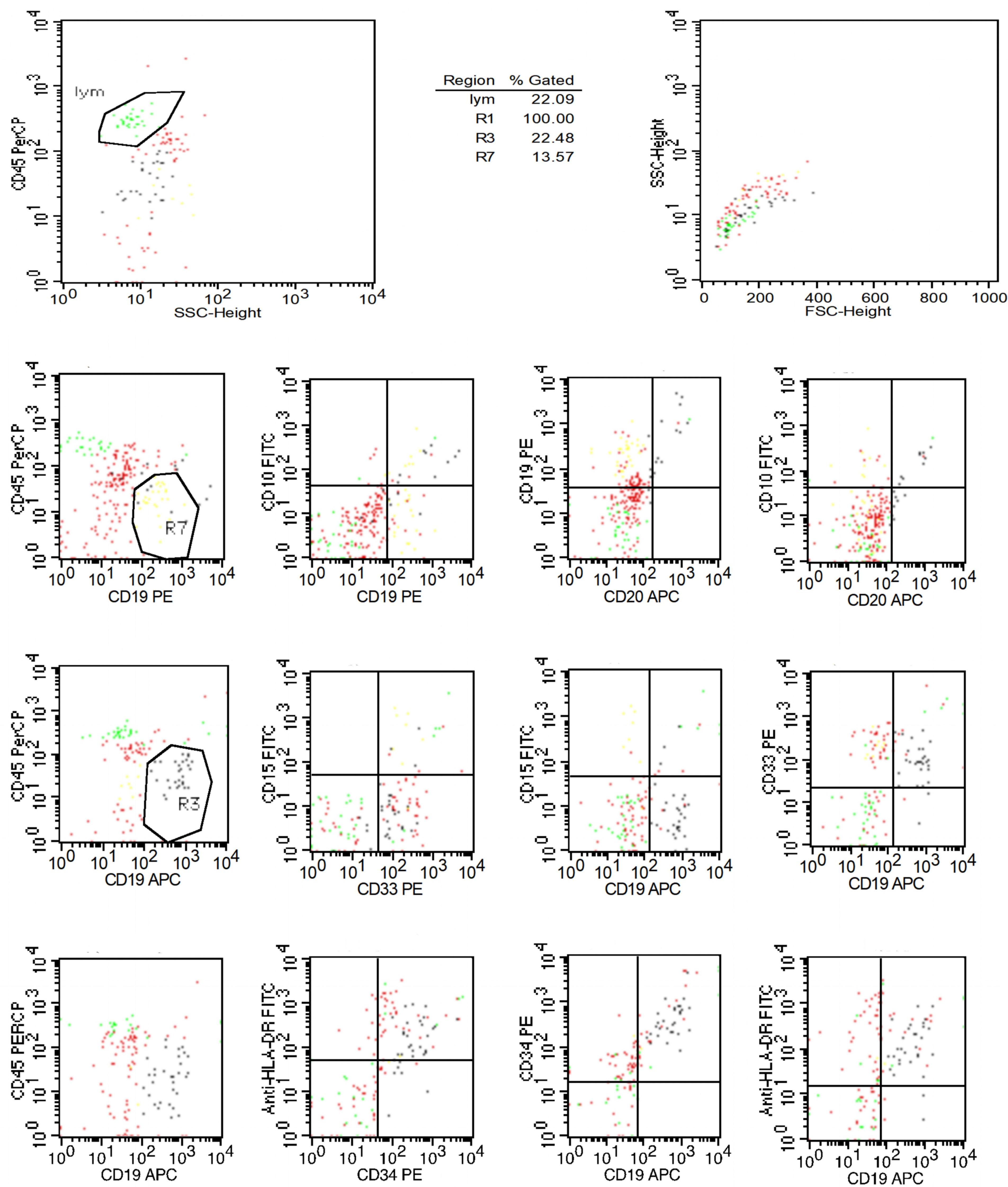

Supplement: Supplementary Figure 1 — The first result of cerebrospinal fluid flow cytometry. [file Image_1.pdf]
